# Forebrain-Specific B-raf Deficiency Reduces NMDA Current and Enhances Small-Conductance Ca^2+^-Activated K^+^ (SK) Current

**DOI:** 10.3390/ijms26157172

**Published:** 2025-07-25

**Authors:** Cornelia Ruxanda, Christian Alzheimer, Fang Zheng

**Affiliations:** Institute of Physiology and Pathophysiology, Friedrich-Alexander-Universität Erlangen-Nürnberg, 91054 Erlangen, Germany

**Keywords:** B-raf, synaptic transmission, NMDA current, small-conductance Ca^2+^-activated K^+^ (SK) channels, hippocampus

## Abstract

B-raf (rapidly accelerated fibrosarcoma) is a crucial player within the ERK/MAPK signaling pathway. In the CNS, B-raf has been implicated in neuronal differentiation, long-term memory, and major depression. Mice with forebrain neuron-specific B-raf knockout show behavioral deficits in spatial learning tasks and impaired hippocampal long-term potentiation (LTP). To elucidate the mechanism(s) underlying diminished synaptic plasticity in B-raf-deficient mice, we performed whole-cell recordings from CA1 pyramidal cells in hippocampal slices of control and B-raf mutant mice. We found that the NMDA/AMPA ratio of excitatory postsynaptic currents (EPSCs) at the Schaffer collateral—CA1 pyramidal cell synapses was significantly reduced in B-raf mutants, which would at least partially account for their impaired LTP. Interestingly, the reduced NMDA component of field postsynaptic potentials in mutant preparations was partially reinstated by blocking the apamin-sensitive small-conductance Ca^2+^-activated K^+^ (SK) channels, which have also been reported to modulate hippocampal LTP and learning tasks. To determine the impact of B-raf-dependent signaling on SK current, we isolated the apamin-sensitive tail current after a strong depolarizing event and found indeed a significantly bigger SK current in B-raf-deficient cells compared to controls, which is consistent with the reduced action potential firing and the stronger facilitating effect of apamin on CA1 somatic excitability in B-raf-mutant hippocampus. Our data suggest that B-raf signaling readjusts the delicate balance between NMDA receptors and SK channels to promote synaptic plasticity and facilitate hippocampal learning and memory.

## 1. Introduction

RAF (rapidly accelerated fibrosarcoma) proteins with serine/threonine kinase activity distribute widely in tissues, including the brain. As effectors for Ras and Rap1 and activators for MEK/ERK, raf proteins stand in a linear kinase cascade of the ERK/MAPK signaling pathway, regulating cell proliferation and survival, development, and cognition [1,2,3,4,5]. Among the three raf isoforms (A-, B-, and C-raf), B-raf is a stronger activator for the ERK/MAPK signaling pathway, with higher MEK binding affinity [6,7]. As mutations in various B-raf domains are associated with human diseases, including mental disorders and tumors, B-Raf is a prime target for molecule-based therapies [8,9,10,11]. In the brain, B-raf is widely distributed in the neuronal soma and the neuritic processes, with its highest immunoreactivity in the hippocampus [12,13]. Using the lentivirus-based rapid gene replacement method in cultured rodent slices and human ESC-induced and iPSC-derived neurons, Lim et al. found that large-scale disease-linked loss- or gain-of-function B-raf mutations induced a wide range of changes in synaptic transmission, closely correlated with the extent of cognitive deficits [14]. With this big data analysis, the authors identified B-raf as the universal signaling effector relaying canonical NMDAR-CaMKII-SynGap-Ras signaling to the MEK-ERK cascade at synapses [14].

Animal studies showed that global B-raf deletion was embryonically lethal [15], and conditional knockout of B-raf in the forebrain glutamatergic neurons from the second postnatal week affected anxiety/depression-like behavior and impaired hippocampal-dependent spatial learning [16,17]. Such conditional B-raf deletion compromised ERK phosphorylation and reduced the magnitude of long-term potentiation (LTP) at the glutamatergic synapses formed by Schaffer collaterals (SC) onto CA1 pyramidal cells [16]. LTP is usually induced by brief, high-frequency tetanic stimulation, which produces a strong temporal summation of excitatory postsynaptic potentials and depolarization in postsynaptic cells. This causes the relief of the Mg^2+^ block of the NMDA receptor (NMDA-R), leading to a strong calcium influx that activates intracellular signaling cascades, including the CaMKII and ERK/MAPK pathways, and ultimately potentiates AMPA receptor (AMPA-R)-mediated synaptic transmission [18,19].

Notably, transient elevations of intracellular Ca^2+^ following NMDA-R activation, Ca^2+^ influx via voltage-dependent Ca^2+^ channels during depolarization, as well as calcium release from intracellular Ca^2+^ stores, also activate small-conductance Ca^2+^-activated potassium channels (SK channels) [20,21,22,23]. SK channels distribute widely along dendrites and soma [24,25]. Especially, SK channels localize strategically to the postsynaptic density of dendritic spines, where they form a Ca^2+^-mediated negative feedback loop with synaptic NMDA-Rs [26,27,28], thus fine-tuning the NMDA-R-dependent synaptic plasticity and learning [29,30,31]. By combining whole-cell patch-clamp recordings and extracellular field potential recordings in mouse brain slice preparation, we identified reduced NMDA current and enhanced SK current in B-raf mutant pyramidal cells as complementary mechanisms for cognitive impairments.

## 2. Results

**Figure 1:**
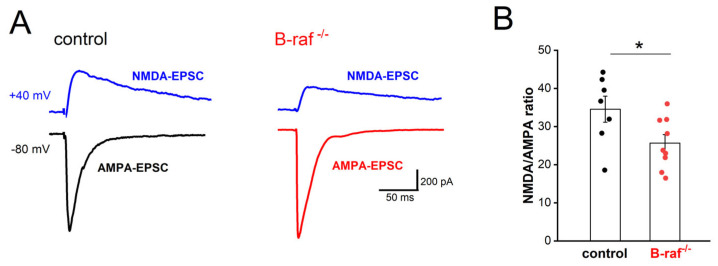
Reduced NMDA/AMPA ratio in B-raf-deficient hippocampus. Whole-cell voltage-clamp recordings were performed in hippocampal CA1 pyramidal cells, in the presence of GABA_A_ receptor antagonist picrotoxin (100 µM), to measure NMDA and AMPA components of excitatory postsynaptic currents (EPSCs) at SC-CA1 pyramidal cell synapses. (**A**) Membrane potential of recorded cells was clamped either at −80 mV to monitor AMPA receptor-mediated EPSCs (AMPA-EPSCs) or at +40 mV for NMDA receptor-mediated EPSCs (NMDA-EPSCs), as depicted by current traces from a control (left) and a B-raf^-/-^ (right) slice. (**B**) Summary of NMDA/AMPA ratio, calculated by dividing the peak amplitude of respective EPSCs in individual cells. Statistical comparison was performed using an unpaired, two-tailed Student’s *t*-test. * *p* < 0.05.

Mice with conditional B-raf deletion showed reduced LTP in the hippocampal CA1 region [16]. Because LTP of the SC-CA1 synapses is NMDA-R dependent, we first examined whether the NMDA component of excitatory transmission is affected by B-raf deficiency. Using whole-cell voltage-clamp recordings, we pharmacologically isolated the CNQX-sensitive AMPA-EPSC (excitatory postsynaptic current; Vh set to −80 mV) and the APV-sensitive NMDA-EPSC (Vh set to +40 mV). As shown in Figure 1, the NMDA/AMPA ratio, calculated by dividing the peak amplitude of NMDA-EPSC by that of AMPA-EPSC, was indeed significantly reduced in B-raf mutant pyramidal cells (control, 34.56 ± 3.40%, n = 7 from 5 mice; B-raf^-/-^, 25.67 ± 2.22%, n = 9 from 5 mice; *p* = 0.039). Such reduction in the NMDA/AMPA ratio at SC-CA1 synapses would at least partially account for their impaired LTP. As an electrophysiological proxy to assess NMDA-R subunit composition, we measured and compared NMDA current kinetics in CA1 pyramidal cells from control and mutant mice on the grounds that changes in NR2 subunit composition would be expected to alter the time course of NMDA currents [32]. Analysis of the NMDA-EPSCs yielded comparable decay time constants in control (207.86 ± 8.25 ms) and B-raf mutants (231.22 ± 9.58 ms; *p* = 0.095). These data would argue against a functionally relevant change in NMDA-R subunit assembly as a consequence of B-raf elimination, but this conclusion remains tentative until being corroborated by molecular evidence in future studies.

**Figure 2:**
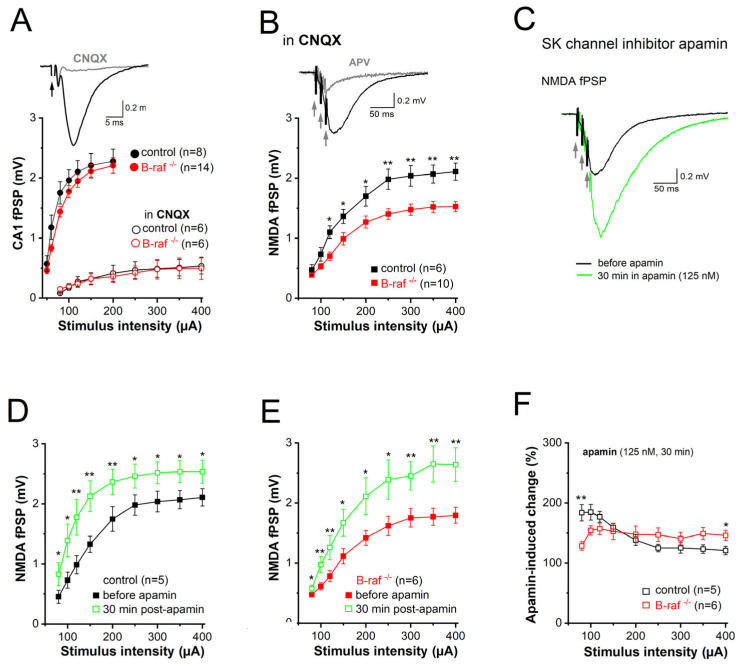
Decreased NMDA receptor-mediated synaptic transmission in CA1 pyramidal cells of B-raf-deficient mice. Field postsynaptic potentials (fPSP) were monitored extracellularly in CA1 stratum radiatum in response to electric stimulation of SC by single stimulus (**A**) or by a burst of 3 stimuli (at 100 Hz; **B**–**F**). (**A**) Input-output (I-O) curves from control and B-raf mutant slices, in the absence and in the presence of AMPA receptor antagonist CNQX (20 µM). Traces on top are recordings from a control slice, illustrating typical field potentials before and during CNQX application (stimulus strength at 80 µA). Arrow indicates truncated stimulus artifact. (**B**) I-O curves for burst stimulus-induced NMDA-fPSPs in the presence of CNQX. Inset depicts suppression of NMDA-fPSP by APV (50 µM). (**C**) Apamin facilitates NMDA-fPSP in a control slice (stimulus strength 80 µA). (**D**,**E**) I-O curves for apamin-enhanced NMDA-fPSPs in control (**D**) and B-raf-deficient slices (**E**), which were normalized individually and pooled as group in (**F**) to reveal relative augmenting effect of apamin as function of stimulus intensity for the two genotypes. Statistical comparisons were performed using ANOVA followed by Tukey’s post-hoc test. * *p* < 0.05; ** *p* < 0.01.

Among the different mechanisms mitigating the NMDA response, one intriguing candidate is the immediate negative feedback regulation of NMDA currents due to the close spatial coupling between NMDA receptors and SK channels in dendritic spines of CA1 pyramidal cells [26]. This well-established concept holds that calcium influx through NMDA receptors activates SK channels, which in turn short-circuit NMDA currents and promote their Mg^2+^ block. To determine whether B-raf-dependent signaling has an impact on SK channel activity in the dendrites, we examined the effect of the selective SK channel blocker apamin on CA1 synaptic transmission in control and B-raf-deficient hippocampi by monitoring field postsynaptic potentials (fPSPs) in CA1 stratum radiatum. Consistent with a previous report ([16], but see [14]), basic synaptic transmission at the SC-CA1 synapse was not changed in B-raf^-/-^ slices, as demonstrated by the nearly perfect superposition of the input-output (I-O) curves that depict peak fPSPs amplitude as a function of stimulus strength for both genotypes (Figure 2A). Given the well-known Mg^2+^ block of NMDA-Rs at resting potentials, CA1 fPSP responses to single SC stimulation should be predominantly mediated by AMPA receptors. In fact, application of the AMPA-R blocker CNQX (20 µM) strongly suppressed fPSPs (Figure 2A, *inset*); even high stimulus intensity (up to 400 µA) failed to produce sizable NMDA responses (Figure 2A). It was only when we used a brief burst-like stimulation paradigm consisting of 3 stimuli @ 100 Hz, which mimics natural firing patterns of hippocampal pyramidal cells during information processing [33], that we elicited a significant CNQX-insensitive fPSP component that was carried by NMDA-Rs, as it was abrogated by the NMDA-R antagonist APV (50 µM; Figure 2B, *inset*). Unlike the AMPA component, the NMDA component of the fPSP was significantly reduced in slices of B-raf^-/-^ mice when compared to their control counterparts (Figure 2B), with maximal responses (to stimulus intensity 400 µA) of 2.11 ± 0.14 mV in controls (n = 7 from 4 mice) and 1.53 ± 0.09 mV in mutants (n = 10 from 5 mice; *p* = 0.003).

Next, we examined the impact of the SK channel inhibitor, apamin (125 nM, 30 min), on the NMDA component in control and mutant slices (Figure 2C). Comparison of the I-O curves before and during apamin superfusion showed a significant increase in NMDA-fPSPs for both genotypes (Figure 2D,E). When we normalized the apamin-induced increase in the NMDA component to the respective pre-drug values, we made the interesting finding that the effect of apamin in slices from transgenic mice was lower for weak stimuli (80 µA) but higher for strong stimuli (400 µA) when compared to control slices (Figure 2F). As a consequence of the latter, apamin equalized the difference in maximal NMDA-fPSPs between genotypes (at stimulus intensity 400 µA: control in apamin, 2.54 ± 0.19 mV, n = 5; B-raf^-/-^ in apamin, 2.64 ± 0.28 mV, n = 6; *p* = 0.780) (Figure 2D,E).

**Figure 3:**
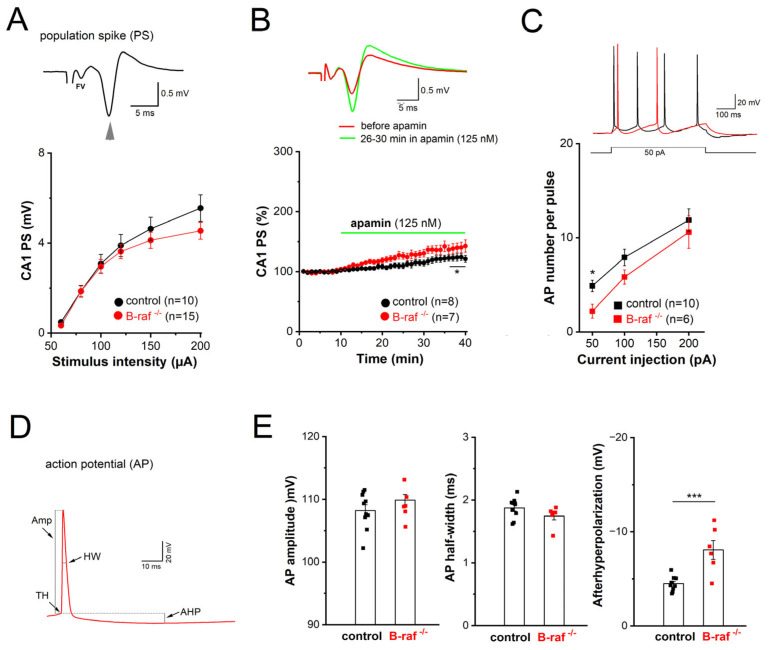
Reduced somatic excitability of CA1 pyramidal cells in B-raf-deficient mice. (**A**,**B**) Population spikes (PS) were recorded in CA1 pyramidal cell layer in response to electric stimulation of SC. (**A**) I-O curves of PS amplitude vs. stimulus intensity in control and B-raf^-/-^ slices. Inset depicts stimulus-induced waveform in control slice. Arrowhead points towards negative peak of PS, and stimulus artifact is truncated. FV, fiber volley. (**B**) Comparison of time course of PS augmentation during apamin application between genotypes. The PS amplitude in each experiment was normalized as percentage of the averaged baseline value (10 min) before apamin application. Inset depicts averaged PS from B-raf-mutant slice before and 26–30 min after onset of apamin administration (stimulus intensity 80 µA). (**C**) Action potentials (APs) of control and B-raf^-/-^ CA1 pyramidal cells, recorded in whole-cell current-clamp mode. AP firing was elicited by depolarizing current steps 500 ms long from membrane potential pre-set to −70 mV, as exemplified by superimposed traces from a control (black trace) and a B-raf-deficient cell (red trace). Plots below depict the relationship between firing rate and size of depolarizing current for the two groups. (**D**) Waveform of first AP after onset of depolarizing current injection (50 pA) in B-raf^-/-^ cells, illustrating the parameters analyzed and summarized in histograms of (**E**). TH threshold, Amp amplitude, HW half-width. Statistical comparisons were performed using one-way ANOVA followed by Tukey’s post-hoc test (**A**–**C**) or an unpaired, two-tailed student’s *t*-test (**E**). * *p* < 0.05; *** *p* < 0.001.

Considering the widespread distribution of SK channels along dendrites and somata of CA1 pyramidal cells, we wondered whether B-raf deficiency would also alter their effect on somatic excitability. For this purpose, we performed field potential and whole-cell recordings in the CA1 pyramidal cell layer to examine population response (termed as population spike, PS) and individual cellular firing of action potentials (APs), respectively. In extracellular PS recordings from the cell body layer, electrical stimulation of SC in stratum radiatum produced a positive-going voltage trajectory reflecting EPSP in the apical dendrites. While still rising, the positive-going trajectory was interrupted by a sharp negative deflection, which is the extracellular correlate of somatic APs once the EPSP became suprathreshold, and the PS was completed with a second positive-going trajectory representing putative feedback inhibition (Figure 3A, *inset)*. Plots of averaged peak PS amplitude against increasing stimulus intensity did not reveal appreciable differences between genotypes (Figure 3A). When the stimulus intensity was adjusted to evoke 30–40% of maximal response, application of apamin (125 nM) after stable baseline led to a gradual increase in PS amplitude, which reached steady-state after 25 min (Figure 3B). Resembling the behavior of the NMDA component in the mutant preparation at maximal stimulus intensity (Figure 2F), PS responses in B-raf-deficient slices displayed higher sensitivity to the SK channel blocker than controls (26–30 min in apamin: control, 118.50 ± 5.23% of baseline value, n = 8 from 4 mice; B-raf^-/-^, 139.57 ± 8.45% of baseline value, n = 7 from 5 mice; *p* = 0.048).

In whole-cell current-clamp recordings, CA1 pyramidal cells from controls and B-raf mutants had comparable resting membrane potentials (control, −65.80 ± 1.90 mV, n = 10 from 4 mice; B-raf^-/-^, −67.20 ± 2.78 mV, n = 6 from 4 mice; *p* = 0.680) and membrane input resistances (measured at −70 mV: control, 177.50 ± 13.79 MΩ; B-raf^-/-^, 183.16 ± 16.41 MΩ; *p* = 0.556). APs were evoked by depolarizing steps (50, 100, and 200 pA) for 500 ms (Figure 3C, inset), with membrane potential pre-set to −70 mV by current injection. As summarized in Figure 3C, B-raf mutant cells fired significantly fewer APs upon small suprathreshold depolarization (50 pA; control, 4.89 ± 0.61 APs per pulse; B-raf^-/-^, 2.20 ± 0.73 APs per pulse; *p* = 0.019), but for stronger depolarizations, the firing frequency vs. depolarizing current relationship (f-I curve) for the two groups appeared to converge gradually (Figure 3C). Further kinetic analysis of individual APs did not reveal alterations in AP amplitude and half-width (Figure 3D,E), nor in voltage threshold (control, −58.05 ± 0.80 mV; B-raf^-/-^, −56.79 ± 1.14 mV; *p* = 0.383) or in maximal rising slope (control, 292.98 ± 18.73 mV/ms; B-raf^-/-^, 316.18 ± 18.24 mV/ms; *p* = 0.352). However, a strong increase in medium after hyperpolarization (mAHP), measured 30–50 ms after AP repolarization, was evident in mutant cells (control, −4.53 ± 0.25 mV; B-raf^-/-^, −8.13 ± 0.99 mV; *p* = 0.0005; Figure 3E).

**Figure 4:**
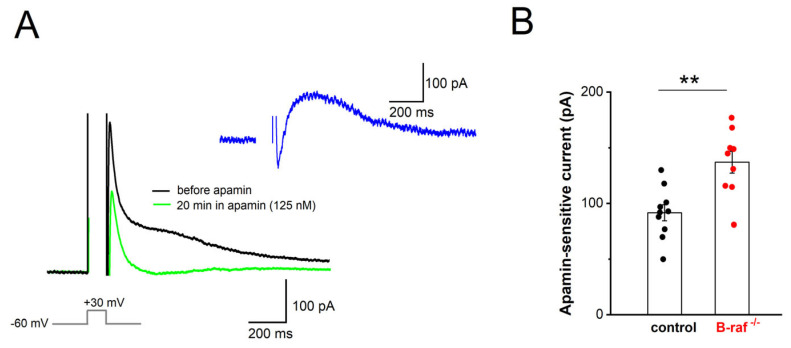
Enhanced contribution of SK current to mAHP in B-raf-deficient mice. (**A**) “Tail current” responses after step depolarization to +30 mV for 100 ms in the absence and presence of apamin (Vh −60 mV; in 1 µM TTX and 5 mM TEA), which, after subtraction, yielded pure apamin-sensitive current (*inset*, blue trace). (**B**) Histogram summarizes apamin-sensitive currents in control and B-raf^-/-^ cells. Statistical comparison was performed using an unpaired, two-tailed Student’s *t*-test. ** *p* < 0.01.

Our findings that, firstly, B-raf deficiency renders PS responses more sensitive to apamin, and that, secondly, the f-I curve in the mutant preparation is attenuated compared to the control condition, might be most parsimoniously explained in terms of an upregulation of SK currents across dendritic and somatic compartments. To substantiate this hypothesis, we performed whole-cell voltage-clamp recordings from CA1 pyramidal cells (Vh −60 mV), using an established protocol to directly examine the currents involved in mAHP generation. In this paradigm, neurons are depolarized to +30 mV for 100 ms, which produces a “tail current” upon returning to −60 mV (Figure 4A). TTX (1 µM) and TEA (5 mM) were used to block Na^+^ channels and other K^+^ channels (such as A-type K^+^ channels and BK channels), respectively. To isolate their SK-mediated component, we measured “tail currents” in the absence and presence of apamin (125 nM, 20–30 min, Figure 4A). Upon subtraction of the currents, the peak amplitude of the SK-dependent tail current was 136.89 ± 9.88 pA in B-raf^-/-^ cells (n = 9 from 4 mice), which was significantly larger than in control cells (91.60 ± 7.24 pA, n = 10 from 5 mice; *p* = 0.002; Figure 4B).

## 3. Discussion

In the present study, we demonstrate that CA1 pyramidal cells in brain slices from B-raf-deficient mice exhibit reduced NMDA currents and stronger SK currents when compared to their counterparts in slices from control mice. The close proximity of NMDA receptors and SK channels at the postsynaptic site enables a local negative feedback mechanism to contain NMDA responses, as the activation of NMDA receptors triggers the subsequent opening of Ca^2+^-activated SK channels [26,27]. In the absence of B-raf signaling, the inverse changes of NMDA and SK currents would operate in concert to impair NMDA-R-dependent LTP at SC-CA1 pyramidal cell synapses, thereby offering a plausible explanation for the poor performance of B-raf-deficient mice in hippocampus-dependent learning and memory tasks [16]. In addition, we found that SK currents are also enhanced in the somatic region of CA1 pyramidal cells, where, by strengthening mAHPs, they serve to control firing rate. We propose that the enhancement of SK current in B-raf-deficient mice has a dual impact on signal processing in CA1 pyramidal cells, affecting both their input (feedback inhibition of NMDA currents) and their output (reduced firing rate due to augmented mAHP).

In the soma and proximal dendrites of hippocampal pyramidal cells, SK channels are activated by Ca^2+^ influx resulting mainly from AP-induced opening of L-type Ca^2+^ channels [34,35]. AP-linked Ca^2+^ transients are maximal in the soma and the proximal apical dendrites and decrease rapidly with distance from the soma [35]. The resultant outflow of K^+^ during SK activation contributes, together with the M-current, to the generation of an AHP of medium duration (mAHP) following AP discharge [34,36,37,38,39]. In addition to showing a significantly enhanced mAHP in the mutant preparation, we obtained direct evidence for a causal involvement of SK channels in the altered mAHP. In whole-cell recordings from CA1 pyramidal cells of B-raf^-/-^ hippocampi, we found that the apamin-sensitive tail current after strong depolarization was markedly upregulated. Based on the functional profile of SK channels, it is well conceivable that the augmented SK current in B-raf-deficient neurons accounts for their reduced excitability, which manifested in our experiments as a decline in firing rate upon suprathreshold depolarization. This view receives support from our population spike (PS) recordings in the pyramidal cell body layer, in which the SK channel blocker apamin produced a much stronger increase in PS amplitude in B-raf mutants than in controls.

In the distal dendrites and spines of hippocampal pyramidal cells, SK channels have been shown to be activated mainly by Ca^2+^ influx through NMDA receptors, which endows them with an immediate impact on the strength of NMDA-dependent synaptic plasticity [26]. Direct measurements of NMDA EPSCs in CA1 pyramidal cells voltage-clamped to +40 mV to record full-blown NMDA currents demonstrated a significant attenuation in the absence of B-raf signaling. Unlike the NMDA component, the AMPA-R-mediated component of excitatory synaptic transmission remained seemingly unaffected in the mutant preparation, as the routinely monitored field synaptic potentials to single stimuli leading mainly to AMPA-R activation did not differ between controls and B-raf mutants.

In our mutant mice, B-raf becomes inactivated in principal forebrain neurons 2–6 weeks after birth, thereby excluding effects on prenatal or early postnatal brain development [17]. In a previous study, adult mice harboring the same conditional B-raf null mutation exhibited deficits in hippocampal LTP, learning, and memory [16]. Thus, reduced NMDA current in conjunction with enhanced SK current may well contribute to their impaired cognitive performance. The question remains, however, whether the electrophysiological and behavioral phenotypes of the conditional B-raf-deficient mice result from impaired ERK/MAPK signaling in the juvenile brain or occur unrelated to any effects on late brain development. A previous study on the affective behavior of this mutant mouse line showed that their low-anxiety phenotype is specifically due to ERK/MAPK deficiency in the juvenile brain, because disruption of ERK/MAPK signaling later in life (9 weeks after birth) failed to alter normal anxiety-like behavior [17]. Whether such a scenario, where suppression of ERK/MAPK during a critical period of late brain development produces a persistent deficit lasting into adulthood, also holds for cognitive functions and their neurophysiological underpinnings remains to be determined in future studies using a similar inducible B-raf knockout approach.

Notably, the decline of the isolated NMDA component in constitutive B-raf-deficient adult brains could be reversed by the SK channel blocker apamin. The augmenting effect of apamin on the NMDA component was much stronger in the mutant than in the control preparation, so that at the highest stimulation strength, the NMDA responses in the two genotypes converged onto the same maximal value, making them virtually indistinguishable. This finding argues for a strong contribution of SK channels to the dampened NMDA response. Whereas B-raf deletion has been shown to affect ERK/MAPK signaling [16,17], the signaling cascade linking alterations in these pathways to the concomitant increase in SK current remains to be elucidated.

In contrast to our findings, a previous study by Lim and colleagues reported that loss-of-function and gain-of-function B-raf mutations produced a wide range of decreases and increases, respectively, in AMPA-R-mediated synaptic transmission in CA1 pyramidal cells, whereas NMDA currents remained unaffected [14]. Based on the results of a systematic replacement of the molecules that constitute the signaling cascade underlying LTP induction, the authors of that study arrived at the conclusion that B-raf serves as a universal signaling effector coupling NMDA-CamKII-SynGap-Ras signaling to the MEK-ERK pathway. In our hands, B-raf deficiency left AMPA responses largely intact, whereas the NMDA component of fPSPs that were evoked by a near-physiological triplet of stimuli [33] in the presence of an AMPA-R blocker exhibited an appreciable decrease in B-raf-deficient slices when compared to the control preparation. How can we explain these apparent discrepancies? The first and foremost difference between their study and our study is the age of the animals. We performed our recordings on acute slices prepared from the brains of adult mice, whereas Lim et al. used preparations from rodents at early life stages, when in our mice *B-raf* had not been targeted yet due to the conditional knockout strategy. Specifically, Lim et al. recorded from CA1 neurons that were prepared from 6- to 7-day-old (postnatal day 6–7) rodents, kept for 8–18 days in culture, and transfected with constitutively active or dominant-negative B-raf mutants using a rapid Sindbis viral expression system (up to 18 h) and a lentivirus-based gene replacement method (within 2–7 days). In addition, they used acute hippocampal slices from young rats (postnatal day 18–28) injected with lentiviral constructs [14]. Apart from general differences between neurons taken in culture shortly after birth and mature neurons in acute slices from the adult brain as a source of inconsistent findings, the fact that the rapid gene replacement method of Lim et al. [14] appeared to have spared NMDA responses suggests that the upregulation of SK current is a late consequence of B-raf deficiency. Thus, our study raises a number of intriguing questions to be followed up in future work. For example, does B-raf signaling directly target SK channels, or is the enhanced SK current a rather slowly developing (mal)adaptive process? How is the increase in SK current achieved: Are new channels produced and inserted in the cell membrane, or is the open probability of existing channels increased? Finally, would administration of an SK channel blocker reverse learning and memory deficits in B-raf-deficient mice and—in the long run—in patients suffering from loss-of-function B-raf mutations?

## 4. Materials and Methods

### 4.1. Animals

Adult (4–7 months old) male and female mice were used for experiments. B-raf knockout (B-raf^-/-^) mice were obtained by breeding floxed B-raf (B-raf^flox^) mice with transgenic CamkIIa-Cre mice [16,17]. The littermates of homozygous B-raf^flox^ without the Cre transgene were used as a control. Such inactivation of the B-raf gene in forebrain principal neurons from two postnatal weeks impaired ERK/MAPK signaling activation [16,17]. Mice were group-housed under standard conditions with a light/dark cycle (7 a.m./7 p.m.) and free access to water and food. All procedures were conducted in accordance with the Animal Protection Law of Germany and the European Communities Council Directive of November 1986/86/609/EEC and with the approval of the local government of Lower Franconia.

### 4.2. Electrophysiological Recordings from Brain Slices

Transversal brain slices (350 µm thick) containing the dorsal hippocampus were prepared from mice under isoflurane anesthesia. Brain slices were cut in ice-cold sucrose-based artificial cerebrospinal fluid (aCSF) containing (in mM) 75 sucrose, 87 NaCl, 3 KCl, 0.5 CaCl_2_, 7 MgCl_2_, 1.25 NaH_2_PO_4_, 25 NaHCO_3_, and 10 D-glucose. Slices were incubated in the same solution for 10 min at 35 °C and then in modified aCSF containing (in mM) 125 NaCl, 3 KCl, 1 CaCl_2_, 3 MgCl_2_, 1.25 NaH_2_PO_4_, 25 NaHCO_3_, and 10 D-glucose at room temperature for at least 2 h before recording. Individual slices were then transferred to a submerged recording chamber perfused with standard aCSF with 1 mM MgCl_2_ and 3 mM CaCl_2_ (unless otherwise stated) at room temperature. All solutions were constantly gassed with 95% O_2_–5% CO_2_.

CA1 field postsynaptic potentials (fPSPs) and population spikes (PS) were recorded in the apical dendrites and the pyramidal cell layer, respectively, with a pipette filled with modified aCSF (in which NaHCO_3_ was replaced by 5 mM HEPES to avoid pH change). A concentric bipolar electrode was inserted into the stratum radiatum to stimulate Schaffer collaterals (SC), with constant current pulses (0.1 ms width). Stimuli were delivered at different intensities, ranging from 60 µA to 200 µA or 400 µA, to construct an input-output (I-O) curve of population responses. Population spikes in the pyramidal cell layer normally three peaks are exhibited, and the amplitude was calculated as the average of the negative peak from the two positive peaks around it. fPSP in the stratum radiatum has a negative peak, and the amplitude was measured as the value of the peak negative from the preceding baseline. In some experiments, to maximize the NMDA component of fPSPs, CNQX (20 µM) was used to block AMPA receptor-mediated fPSPs, and a burst pattern (3 stimuli at 100 Hz) was used to evoke NMDA receptor-mediated fPSPs. The effect of the SK channel blocker apamin (125 nM) on PS and fPSP was tested with stimulus intensity set to induce 30–40% of maximal field potentials and was continuously monitored until steady state was reached (normally 20–30 min of drug wash-in). At this time point, measurements of I-O curves for field potentials were repeated. As apamin is hard to wash out from brain slices, the effect of apamin was quantified by comparing the values between baseline (before apamin) and the maximal effect (26–30 min in apamin) in the same slices, and data from each genotype were grouped.

Whole-cell recordings were performed from visually identified CA1 pyramidal cells in the pyramidal cell layer under voltage-clamp mode or current-clamp mode. For experiments to evaluate the NMDA/AMPA ratio among excitatory synaptic transmission in voltage-clamped cells, the patch pipettes were filled with (in mM) 130 CsCl, 2 MgCl_2_, 2 Na_2_ATP, 0.3 Na_3_GTP, 5 QX-314, 5 HEPES, and 5 EGTA (pH 7.3). Excitatory postsynaptic current (EPSC) was evoked by stimulus via a bipolar electrode in stratum radium in the presence of GABA_A_ receptor antagonist picrotoxin (100 µM). The stimulus strength was set at the beginning of each experiment so that the EPSC peak was around 500 pA at a holding potential of −80 mV. The AMPA receptor-mediated EPSC (AMPA-EPSC) was isolated by subtracting EPSC in the presence of CNQX (20 µM) from that in its absence. The NMDA receptor-mediated EPSC (NMDA-EPSC) was recorded at +40 mV in the presence of CNQX and subtracted from that in the presence of DL-APV (50 µM). Glycine (10 µM) was added into aCSF to ensure a saturating concentration of glycine for NMDA receptors.

To assess somatic SK channel activity and pyramidal cell excitability, the recording pipette contained (in mM) 135 K-gluconate, 4 NaCl, 10 KCl, 2 Na_2_ATP, 0.3 Na_3_GTP, and 5 HEPES (pH 7.3), and the recordings were performed in aCSF with 2 mM CaCl_2_ and 1 Mg MgCl_2_. Tail currents in voltage-clamped pyramidal cells (Vh −60 mV) were evoked by a depolarizing command to +30 mV for 100 ms, thereby triggering sufficient calcium current to activate SK channels, in the presence of TTX (1 µM, to block sodium current) and tetraethylammonium (TEA, 5 mM; to block other potassium currents, e.g., voltage-dependent K^+^ channel, large conductance calcium-activated potassium channels, or BK channels). Recordings in the absence and presence of the selective SK channel blocker apamin (125 nM, added to inflowing bath solution) were used to isolate and quantify peak SK currents, which were determined about 100 ms after stepping back to −60 mV). Further experiments to examine the excitability of CA1 pyramidal cells were conducted in whole-cell current-clamp mode. To facilitate comparison between control and B-raf mutants, action potentials (APs) were evoked by depolarizing current pulses (50, 100, and 200 pA; 500 ms duration), with membrane potential set to −70 mV by current injection. AP properties were analyzed for the first AP in response to a 50 pA depolarizing step, including voltage threshold, rise time and amplitude of upstroke, and half-width. The medium afterhyperpolarization (mAHP), which partially involves SK channel activity [36,37], was measured 30–50 ms after repolarization.

All potentials were corrected for liquid junction potential (10 mV). Series resistance in the whole-cell voltage-clamp configuration was 10–20 MΩ and compensated by 60–80%. Signals were filtered at 2 kHz (for extracellular and whole-cell voltage-clamp recordings) and 6 kHz (for whole-cell current-clamp recordings) and sampled at 20 kHz using a Multiclamp 700B amplifier, in conjunction with a Digidata 1440A interface and pClamp10.2 software (all from Molecular Devices, Sunnyvale, CA, USA). MiniDigi 1A and AxoScope 10.2 were used for low-resolution scope recording, sampled at 1 kHz. To obey the ‘3R’ rule of animal research and to ensure adequate power to detect the effect of treatment, individual parameters were sampled from 4 to 7 mice for each group, with sample sizes of 5–14 slices based on our electrophysiology expertise. Unless otherwise stated, drugs and chemicals were obtained from Tocris Bioscience (Bio-techne GmbH, Wiesbaden, Germany) and Sigma-Aldrich Chemie GmbH (Steinheim, Germany).

### 4.3. Statistical Analysis

Data analysis was performed off-line with Clampfit 10.2 (Molecular Devices, Sunnyvale, CA, USA). Data are expressed as means ± SEM. OriginPro 2018G (OriginLab Corporation, Northampton, MA, USA) was used for statistics and graphs. The Shapiro–Wilk test was used to assess the normality of data distribution, and the null hypothesis was accepted when the *p*-value was larger than 0.05. Statistical comparisons were performed using unpaired or paired Student’s *t*-tests and one-way or two-way analysis of variance (ANOVA) followed by Tukey’s post-hoc test, as appropriate. Significance was assumed for *p* < 0.05.

## Data Availability

Data from this study are available from the corresponding author upon reasonable request.

## Abbreviations

The following abbreviations are used in this manuscript: aCSF, artificial cerebrospinal fluid; AP, action potential; AMPA-R, AMPA receptor; B-raf, type B isoform of rapidly accelerated fibrosarcoma; ERK/MARK, extracellular signal-regulated kinase/mitogen-activated protein kinase; I-O, input-output curve; LTP, long-term potentiation; EPSC, excitatory postsynaptic current; fPSP, field postsynaptic potential; mAHP, medium afterhyperpolarization; NMDA-R, NMDA receptor; PS, population spike; SC, Schaffer collaterals; SK, small-conductance Ca^2+^-activated K^+^ channels; TEA, tetraethylammonium; TTX, tetrodotoxin.
